# Supplementary material for: Respiratory complex III2 assembles complex I via toxic intermediate in mitochondrial disease
Source: bioRxiv. 2025 Jun 18:2025.06.17.660237. Preprint. [Version 1] doi: 10.1101/2025.06.17.660237 (PMC12262534; doi:10.1101/2025.06.17.660237)
Supplement: 1 [file NIHPP2025.06.17.660237V1-supplement-1.pdf]

bioRxiv preprint doi: https://doi.org/10.1101/2025.06.17.660237; this version posted June 18, 2025. The copyright holder for this preprint (which was not certified by peer review) is the author/funder, who has granted bioRxiv a license to display the preprint in perpetuity. It is made available under aCC-BY 4.0 International license.

Supplementary Materials

Material and Methods

Supplementary Text

Figs. S1 to S12

Tables S1 to S3

References (64-65)

Material and Methods

Animals and genotype analysis

All animal protocols were approved by the Institutional Animal Care Use Committee at the University of California, Davis and were also in accordance with the NIH guidelines for the Care and Use of Laboratory Animals. The NDUFS4$^{+/-}$ mouse strain on a C57BL/6J background was provided by the Jackson Laboratory (Bar Harbor, ME) and purchased for in-house breeding. To produce constitutive NDUFS4 knockout mice, mice heterozygous for the NDUFS4 knockout (NDUFS4$^{+/-}$) were bred together. Wild-type littermates (NDUFS4$^{+/+}$) were used as controls and heterozygotes (NDUFS4$^{+/-}$) were also assessed in parallel. Male and female mice were housed in polycarbonate cages starting at 21 days of age on a 12-hour light/dark cycle. Body weights were monitored weekly. Mice were provided DI water and a standard rodent chow *ad libitum* (Teklad 2018, Inotivco). Mice were provided with Mouse Igloos (Bio-Serv) to provide additional shelter. Mice were euthanized by carbon dioxide overdose at 43 days of age and intra-cardiac perfusion performed with 5 mL of phosphate buffered saline before removal of target organs. We prepared genomic DNA from ear clip samples collected from the mice between 12-20 days of age by using a HotShot DNA extraction (using 0.5M NaOH and Tris HCl pH 7.0 solutions), and PCR-based genotyping was completed using the published primer sets (*24*) for wild type and knockout alleles (Integrated DNA Technologies).

Mitochondrial purification

Liver tissues were excised from wild-type and NDUFS4 knockout C57BL/6J mice and rinsed in PBS before blotting them dry. The tissues were then flash frozen in liquid nitrogen and stored at -70 ˚C. All steps were performed at 4˚C with pre-chilled materials. Mitochondria were isolated from the knockout and wildtype tissues as described previously (*64*). Briefly mouse livers were thawed at 4 ˚C before being minced into 1 mm pieces and homogenized in 10 mL buffer AT (0.075 M sucrose, 0.225 M sorbitol, 1 mM EGTA, 0.1% fatty acid-free bovine serum albumin (BSA), and 10 mM Tris-HCl, pH 7.4, supplemented with SIGMAFAST Protease Inhibitor

bioRxiv preprint doi: https://doi.org/10.1101/2025.06.17.660237; this version posted June 18, 2025. The copyright holder for this preprint (which was not certified by peer review) is the author/funder, who has granted bioRxiv a license to display the preprint in perpetuity. It is made available under aCC-BY 4.0 International license.

Cocktail Tablets) per gram of tissue using a Potter-Elvehjem homogenizer fitted with a Teflon pestle for 50 strokes. The homogenate was spun down at 1,000 g for 5 min. The supernatant from that spin was transferred to eight 1.5 mL Eppendorf microcentrifuge tubes and spun at 15,000 g for 2 min. The supernatant was removed, leaving only the brown mitochondrial pellet. Two mitochondrial pellets were combined dividing the number of tubes in half and were resuspended in 1.5 mL of medium AT using a pipette. The combined pellets were spun down at 15,000 g for 2 min. The process was repeated until there was only 1 pellet in one tube. The supernatant was removed from the final pellet and the pellet was considered crude mitochondria and stored at -70 ˚C.

Mitochondrial membrane wash

All steps were performed at 4˚C with pre-chilled materials. Unfrozen mitochondria from the mitochondrial purification step were homogenized in 10 mL MilliQ water per gram of mitochondria using a Dounce glass homogenizer for 100 strokes. Potassium chloride was added to final concentration of 0.15 M and sample was homogenized again for 100 strokes. The homogenate was centrifuged at 43,667 g for 50 min. The pellet was resuspended in 18 mL Buffer M (20 mM Tris pH 7.4, 50 mM NaCl, 1 mM EDTA, 10% (v/v) Glycerol, 2 mM DTT, 0.002% PMSF) per gram of mitochondria starting material for and homogenized again for 100 strokes. The homogenate was centrifuged at 28,302 g for 50 min. The pellet was resuspended in 3 mL Buffer M (20 mM Tris pH 7.4, 50 mM NaCl, 1 mM EDTA, 10% glycerol, 2 mM DTT, 0.002% PMSF) per gram of mitochondrial starting material and homogenized again for 100 strokes. Protein concentration of the final membranes was calculated using a Pierce BCA assay kit and was diluted to final concentration of 10 mg/ml in 30% (v/v) glycerol for storage at -70 ˚C.

Supercomplex purification

Mouse liver washed mitochondrial membranes were thawed on ice. Supercomplex were isolated by tumbling for 45 min at 4 °C with digitonin at a 4:1 (w/w) ratio and 1% (w/v) concentration in Buffer MX (30 mM HEPES, 150 mM potassium acetate, 10% v/v glycerol, 1 mM EDTA and 0.002% PMSF). The sample was centrifuged at 15,973 *g* for 45 min at 4 °C and the supernatant was kept. The supernatant was concentrated using (100 kDa cutoff) centrifugal concentrators to a final volume of 500 µl (knockout) and 250 µl (wildtype) to inject onto the size exclusion chromatography (SEC) Superose 6 increase 10/300 GL column pre equilibrated with SEC buffer (30 mM HEPESpH 7.8, 150 mM potassium acetate, 1 mM EDTA, 0.005% (w/v) GDN). Fractions were run on a 3-12% BN-page and subjected to CI-in-gel activity assay.

bioRxiv preprint doi: https://doi.org/10.1101/2025.06.17.660237; this version posted June 18, 2025. The copyright holder for this preprint (which was not certified by peer review) is the author/funder, who has granted bioRxiv a license to display the preprint in perpetuity. It is made available under aCC-BY 4.0 International license.

Fractions showing CI activity were pooled for both the knockout and wildtype samples. The pooled fractions were concentrated using (100 k Da cutoff) centrifugal concentrators and protein concentration was measured using a Pierce BCA assay kit and diluted to ~5 mg/ml in 0.05 % digitonin in Buffer MX to freeze cryoEM grids.

Cryo-EM grid preparation and data collection

For the WT, data was collected from two different cryoEM grids. Three microliters of 6 mg/mL fractions from Superose 6 increase 10/300 GL column were applied onto a C-Flat 1.2/1.3 20 nm carbon on 300 mesh gold grid, glow discharged at 30 mA for 20 seconds. The grids were incubated with sample for 10 seconds pre-blotting at 20 ˚C and 90% humidity. One grid was blotted for 7 seconds and the other for 8 seconds before plunge-freezing into liquid ethane. A total of 16,310 movies were collected using EPU on a 300 kV Titan Krios with a pixel size of 0.86 Å/pixel. A dose of 50.5 electrons/$Å^2$ with a 1.68 s exposure time was fractionated into 40 frames for each movie.

For the $S4^{KO}$, data was collected from three different cryoEM grids. Three microliters of 6 mg/ml fractions from Superose 6 increase 10/300 GL column were applied onto a C-Flat 1.2/1.3 20 nm carbon on 300 mesh gold grid, glow discharged at 30 mA for 20 seconds. The grids were incubated with sample for 10 seconds pre-blotting at 20 ˚C and 90% humidity. Two grids were blotted for 10 seconds and the other for 6 seconds before plunge-freezing into liquid ethane. A total of 17,190 movies were collected using EPU on a 300 kV Titan Krios with a pixel size of 0.86 Å/pixel. A dose of 49.78 electrons/$Å^2$ with a 1.63 s exposure time was fractionated into 40 frames for each movie.

Cryo-EM image pre-processing for knockout and wildtype sample

The raw movies were motion-corrected using MotionCor2 and per-micrograph contrast transfer function (ctf) estimation was calculated using the CTFFIND4.1 in Relion 4.1.0. Using Warp (*65*), micrographs were curated to remove 556 in the WT and 958 in the $S4^{KO}$ datasets. Particles were picked using a trained model. The initial 1,215,504 WT and 1,424,255 $S4^{KO}$ picked particles were extracted in Warp with 600 $pixel^2$ boxes and imported into cryoSPARC v4.4.1. Iterative 2D classification, 3D ab initio reconstruction, and 3D refinement were performed initially in CryoSPARC.

In the WT sample, 117,010 good particles were obtained after the final round of 2D classification. 3D *ab initio* and 3D classification resulted in 61,454 particles corresponing to SC $I+III_2$ and 31,935 to the respirasome. Homogeneous refinement and non-uniform refinement of

bioRxiv preprint doi: https://doi.org/10.1101/2025.06.17.660237; this version posted June 18, 2025. The copyright holder for this preprint (which was not certified by peer review) is the author/funder, who has granted bioRxiv a license to display the preprint in perpetuity. It is made available under aCC-BY 4.0 International license.

each of the classes resulted in reference map of 3.5 Å. The particle set was then transferred back into Relion 4.1.0 for global search, CTF refinement, Bayesian polishing and local searches resulting in a final map of 3.0 Å for SC I+III_2 and 3.1 Å for the respirasome. These maps were used for initial model building in coot and refinement in phenix.

In the $S4^{KO}$ sample, 77,586 good particles were obtained after the final round of 2D classification. 3D *ab initio* and 3D classification resulted in 88,714 particles in 18 classes that corresponded to 14,547 SC I+III_2, 18,452 to the respirasome, 8,007 to SC $I_{Q/P}$+III_2 and 8,037 to $R_{Q/P}$. Lastly, heterogenous refinement of particles missing the N-module yielded a class of 3,000 particles without peripheral arm. Homogeneous refinement and non-uniform refinement of each of the classes resulted in reference map of 3.4 Å. The particle set was then transferred back into Relion 4.1.0 for global search, CTF refinement, Bayesian polishing and local searches resulting in a final map of 3.3 Å for SC I+$III_{2,}$ 3.5 Å for the respirasome, 3.8 Å for N-less SC I+III_2 and 3.9 Å for $R_{Q/P}$. These maps were used for initial model building in coot and refinement in phenix.

Model building and refinement

Model building was performed in Coot and refinements in Phenix 1.21. For the WT, CI structure from murine (6ZR2) and SC III_2+IV structure from murine (7O3C) were docked into our structures. For the $S4^{KO}$, CI structure from murine (8CA5) and SC III_2+IV structure from murine (7O3C) were docked into our structures. The models, were manually inspected, adjusted, and rebuilt where necessary to generate our model.

Blue native PAGE

Mouse liver mitochondrial membranes were solubilized using 1% digitonin for 45 min and centrifuged at 16,130 g for 30 min. The supernatant was concentrated, and 40 µg of total protein were loaded on Bio-Rad 4-15% Mini-PROTEAN TGX Precast Gels. The gel was run for 30 minutes at 150 V in buffer containing 0.02% Coomassie Brilliant Blue G. After, the gel was run for 1 hour and 30 minutes at 200 V in a buffer containing $1/10^{th}$ of the Coomassie Brilliant Blue G buffer. In-gel complex I activity assays were performed using 150 µM NADH and 1.5 mg/mL Nitrotetrazolium Blue chloride (NTB). In-gel complex IV activity assays were performed using 10 mM phosphate buffer pH 7.4, 50 mM NaCl, 0.5 mg/mL 3,3'-diaminobenzidine (DAB) and 80 µM cytochrome *c*.

Western blotting

bioRxiv preprint doi: https://doi.org/10.1101/2025.06.17.660237; this version posted June 18, 2025. The copyright holder for this preprint (which was not certified by peer review) is the author/funder, who has granted bioRxiv a license to display the preprint in perpetuity. It is made available under aCC-BY 4.0 International license.

Protein complexes were separated using the blue native PAGE methods described above. The proteins were transferred to polyvinylidene difluoride (PVDF) membranes using a Transblot Turbo Transfer System (Bio-Rad). The PVDF membrane was blocked in 5% (w/v) nonfat dry milk in tris-buffered saline (TBS) overnight. The membranes were washed twice for 10 min each in 0.05% Tween 20 in TBS (TBST). Following the washes, the membranes were incubated in the appropriate primary antibody dissolved in 5% (w/v) nonfat dry milk in TBST for 2 hours at room temperature. The membranes were washed four times for 10 min each in TBST. After the washes, the membranes were incubated for 1 hour at room temperature with the appropriate HRP-conjugated secondary antibody dissolved in TBST. Then, the membranes were washed four times for 10 min each in TBST. A final wash was performed in TBS to remove Tween 20 from the membrane surface. Immunoreactivity was detected by a Prometheus Protein Biology Products ProSignal Femto kit (Genese Scientific) and analyzed by the Lumenescent Image Analyzer (Image Quant LAS400). Protein immunodetection was performed using the primary antibodies: anti-NDUFS6 (ab195807, Abcam), anti-NDUFS4 (ab139178, Abcam), anti-NDUFA10 (PA5-22348, Invotrogen), anti-NDUFA9 (459100, Abcam), anti-CORE Protein I (ab110252, Abcam), anti-NDUFS1(PA5-22309, Invitrogen). The secondary antibodies used were: goat anti-rabbit IgG (ab6721), and goat anti-mouse (AP181P, EMD Millipore).

Complex I Activity

Complex I activity was measured from wildtype and knockout murine liver mitochondrial membranes in reaction buffer (20 mM HEPES pH 7.4, 50 mM NaCl, 10% (w/v) glycerol, 0.1% (w/v) CHAPS, 0.25 mg/mL 4:1 Asolectin:Cardiolipin, 1 mg/mL BSA, 100 μM Decylubiquinone) at 200 mg by measuring NADH oxidation at 340 nm in 4.5 mL cuvettes at room temperature using the Cary 60 UV-Vis (Agilent). Mitochondrial membranes were mixed with reaction buffer using a stir bar to a final volume of 1.5 mL. The reaction was initiated by the addition of 150 μM NADH and Rotenone (1 μM) was used to inhibit CI and show specificity. Measurements of the initial rates were done in triplicates, averaged and normalized. A CI assay as described above was used to measure the stability of CI over time after extraction in either Digitonin or DDM.

Complex IV Activity

Complex IV activity was measured from wildtype, heterozygote, and knockout murine liver and heart mitochondrial membranes in reaction buffer (20 mM HEPES pH 7.4, 50 mM NaCl, 10% (w/v) glycerol, 0.1% (w/v) CHAPS, 0.25 mg/mL 4:1 Asolectin:Cardiolipin, 1 mg/mL BSA) at 200 mg by measuring oxygen consumption using an Oxygraph+ (Hansatech Instruments Ltd). The

bioRxiv preprint doi: https://doi.org/10.1101/2025.06.17.660237; this version posted June 18, 2025. The copyright holder for this preprint (which was not certified by peer review) is the author/funder, who has granted bioRxiv a license to display the preprint in perpetuity. It is made available under aCC-BY 4.0 International license.

reaction buffer was added to the Oxygraph+ chamber with cytochrome *c* (100 μM) and the mitochondrial membranes and was constantly mixed using a stir bar. The reaction was initiated by the addition of TMPD (300 mM) and ascorbate (3 mM). Sodium Azide (1 mM) was used to inhibit complex IV. Measurements of the oxygen concentration were done in a minimum of triplicates, averaged and normalized.

Complex II Activity

Complex II activity was measured from wildtype, heterozygote, and knockout murine liver mitochondrial membranes in reaction buffer (50 mM HEPES pH 8.0, 0.1 mM EDTA, 1 mg/mL BSA, 0.25 mg/mL 4:1 Asolectin:Cardiolipin, 4 μM KCN, 1 μM Rotenone, 2 μM Antimycin, 100 μM Decylubiquinone) at 200 mg by measuring DCPIP reduction at 600 nm using the Cary 60 UV-Vis (Agilent). Measurements were made in 4.5 mL cuvettes at room temperature at a final volume of 1.5 mL with constant stirring using a stir bar. Membranes were added to the buffer and were allowed to equilibrate, DCPIP (100 μM) was added, and the reaction was initiated by the addition of succinate (100 μM). Oxaloacetate (200 μM) was used to inhibit complex II to show specificity. Measurements of the initial rates were done in a minimum of triplicates, averaged and normalized.

NEM Assay

The NEM assay was performed in 96 well plates for wildtype and knockout murine liver and heart mitochondrial membranes in reaction buffer (20 mM HEPES pH 7.4, 50 mM NaCl, 10% (w/v) glycerol, 0.1% (w/v) CHAPS, 0.25 mg/mL 4:1 Asolectin:Cardiolipin, 1 mg/mL BSA, 100 μM Decylubiquinone) at 300 μg for the knockout and 30 μg for the wildtype by measuring NADH reduction at 340 nm. Mitochondrial membranes from wildtype and knockout hearts and livers were incubated at 37 ˚C for 30 (wildtype) and 15 (knockout) minutes or left as is. 5 μM pre NADH or an equivalent amount of buffer was added to the corresponding sample and mixed by pipetting. 30 seconds after the addition of pre NADH or buffer 2 mM NEM or water was added to the corresponding well and mixed by pipetting. The plates were covered and incubated at room temperature for 20 minutes. The reaction was started by the addition of 200 μM NADH and NADH oxidation was measured at 340 nm using a Molecular Devices (San Jose, CA) Spectramax M2 spectrophotometer. Measurements of the initial rates were done in triplicates, averaged and normalized.

Supplementary Figures

bioRxiv preprint doi: https://doi.org/10.1101/2025.06.17.660237; this version posted June 18, 2025. The copyright holder for this preprint (which was not certified by peer review) is the author/funder, who has granted bioRxiv a license to display the preprint in perpetuity. It is made available under aCC-BY 4.0 International license.

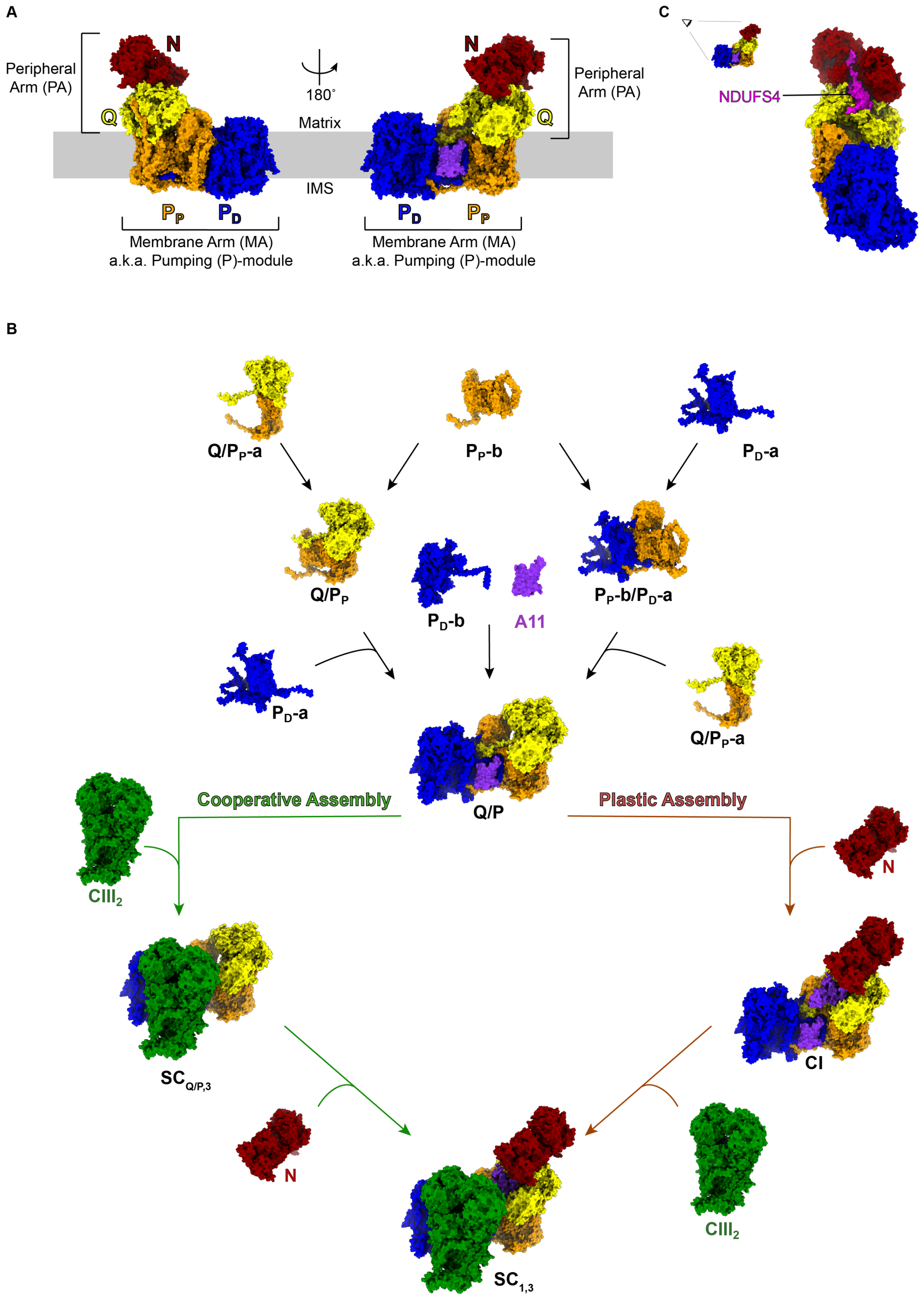

1

bioRxiv preprint doi: https://doi.org/10.1101/2025.06.17.660237; this version posted June 18, 2025. The copyright holder for this preprint (which was not certified by peer review) is the author/funder, who has granted bioRxiv a license to display the preprint in perpetuity. It is made available under aCC-BY 4.0 International license.

Figure S1. Modular nature of CI structure. (**A**) Mouse WT CI structure colored by module with the different arms of the complex indicated. (**B**) Simplified modular assembly pathway for CI comparing the cooperative assembly model vs. assembly first plasticity model. (**C**) Location of subunit NDUFS4.

bioRxiv preprint doi: https://doi.org/10.1101/2025.06.17.660237; this version posted June 18, 2025. The copyright holder for this preprint (which was not certified by peer review) is the author/funder, who has granted bioRxiv a license to display the preprint in perpetuity. It is made available under aCC-BY 4.0 International license.

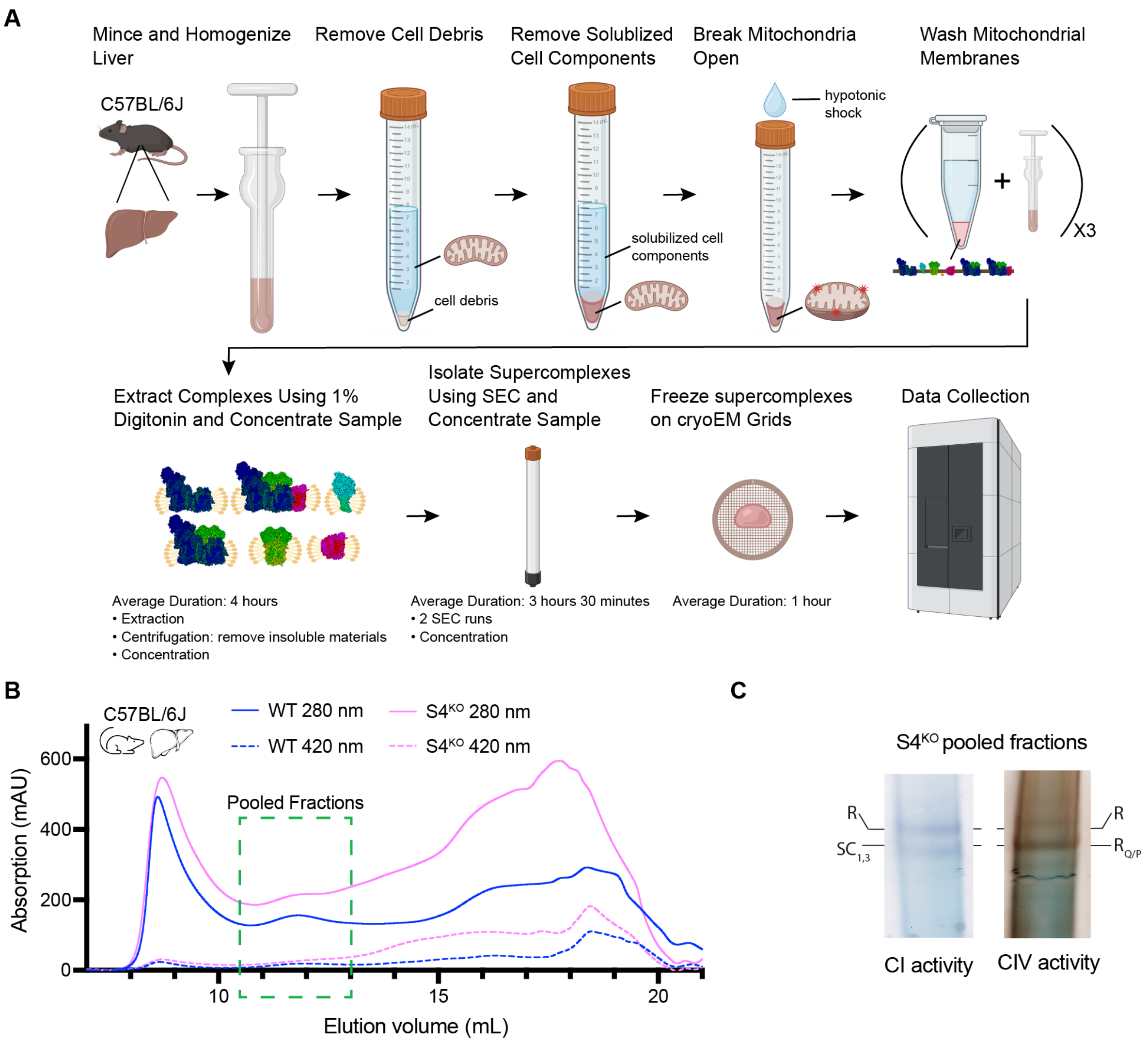

Figure S2. Biochemical preparation of SC samples. (**A**) Schematic showing the steps from tissue homogenization to sample freezing on grids. (**B**) Superose 6 Increase 10/300 size exclusion column (SEC) chromatogram of 1% digitonin (w/v) extracted mitochondrial membranes from liver. WT: NDUFS4$^{+/+}$ and S4^{KO}: NDUFS4$^{-/-}$. The green dotted box indicates the fractions that were pooled and concentrated for grid freezing. (**C**) Blue-native PAGE (BN-PAGE) CI (left) and CIV (right) in-gel activity assays of S4^{KO} pooled fractions from (**B**). Labels: R: Respirasome; $SC_{1,3}$: Supercomplex I+III_2; $R_{Q/P}$: Respirasome containing CI Q/P intermediate.

bioRxiv preprint doi: https://doi.org/10.1101/2025.06.17.660237; this version posted June 18, 2025. The copyright holder for this preprint (which was not certified by peer review) is the author/funder, who has granted bioRxiv a license to display the preprint in perpetuity. It is made available under aCC-BY 4.0 International license.

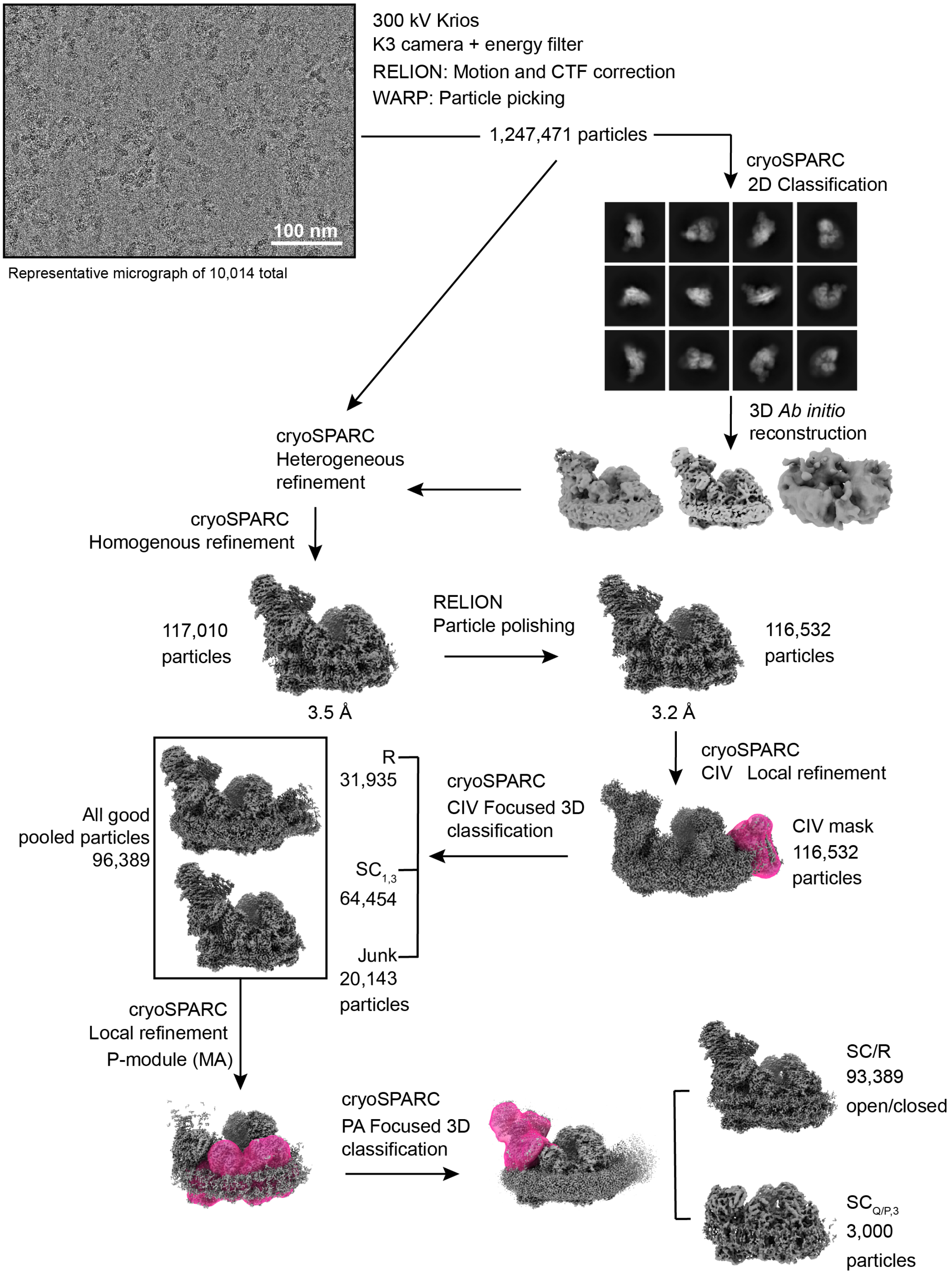

Figure S3. **WT data processing overview**. Data were collected at the SLAC-Stanford CryoEM Center on a 300 kV Titan Krios microscope (TEM-beta) with Gatam K3 camera (see also Table S1). After removal of poor-quality micrographs a total of 10,014 images were processed

bioRxiv preprint doi: https://doi.org/10.1101/2025.06.17.660237; this version posted June 18, 2025. The copyright holder for this preprint (which was not certified by peer review) is the author/funder, who has granted bioRxiv a license to display the preprint in perpetuity. It is made available under aCC-BY 4.0 International license.

(representative micrograph shown), from which 1,247,471 particles were initially picked using WARP (*65*). The particles were sorted using a cryoSPARC heterogeneous refinement with 3D *ab initio* reconstructions from 2D classification as inputs. The heterogenous refinement yielded 117,010 particles that were imported into RELION. The particle set was cleaned further in RELION and after particle polishing 116,532 particles were imported back into cryoSPARC. A CIV local refinement and CIV focused 3D classification was performed to sort R, $SC_{1,3}$ and junk particles. 96,389 good particles were obtained, and a MA local refinement was performed followed by a PA focused 3D classification to sort N-module containing from N-less particles. 3,000 $SC_{Q/P,3}$ particles were obtained and the 93,389 $SC_{1,3}$ and R particles were further sorted in open and closed states. R: Respirasome; $SC_{1,3}$: Supercomplex I+III_2; MA: membrane arm; PA: peripheral arm; $SC_{Q/P,3}$: Supercomplex with CI Q/P intermediate plus $CIII_2$ (see Table S3).

bioRxiv preprint doi: https://doi.org/10.1101/2025.06.17.660237; this version posted June 18, 2025. The copyright holder for this preprint (which was not certified by peer review) is the author/funder, who has granted bioRxiv a license to display the preprint in perpetuity. It is made available under aCC-BY 4.0 International license.

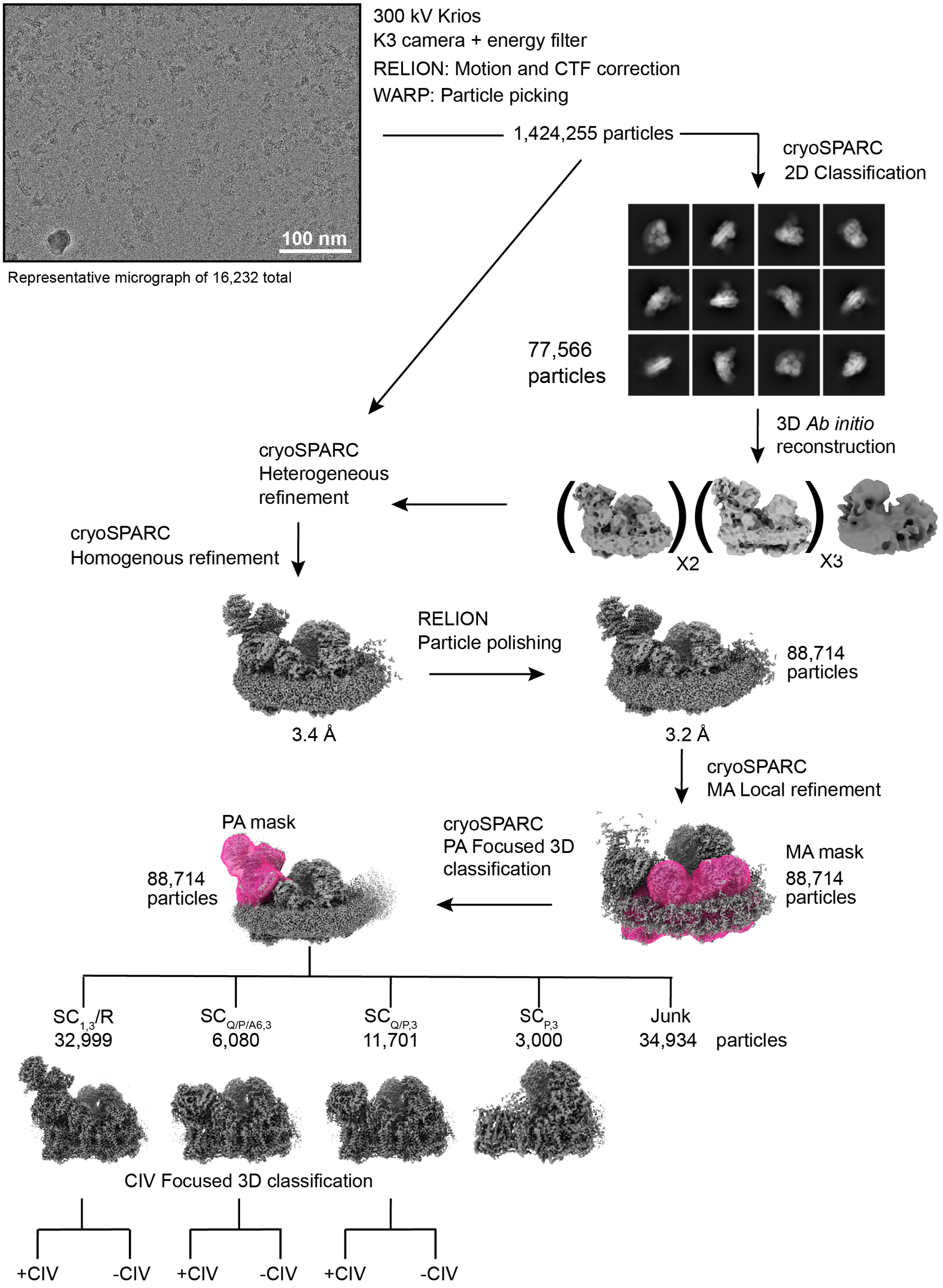

Figure S4. S4^{KO} data processing overview. Data were collected at the SLAC-Stanford CryoEM Center on a 300 kV Titan Krios microscope (TEM-beta) with Gatam K3 camera (see

bioRxiv preprint doi: https://doi.org/10.1101/2025.06.17.660237; this version posted June 18, 2025. The copyright holder for this preprint (which was not certified by peer review) is the author/funder, who has granted bioRxiv a license to display the preprint in perpetuity. It is made available under aCC-BY 4.0 International license.

also Table S1). A total of 16,232 images were processed (representative micrograph shown), from which 1,424,255 particles were initially picked using WARP (*65*). The particles were sorted using a cryoSPARC heterogeneous refinement with 3D ab initio reconstructions from 2D classification as inputs. The heterogenous refinement yielded 95,628 particles that were imported into RELION. The particle set was cleaned further in RELION and after particle polishing 88,714 particles were imported back into cryoSPARC. A MA local refinement and PA focused 3D classification was performed to separate N-module containing, N-less particles and $SC_{P,3}$ particles. The $SC_{1,3}$/R, $SC_{Q/P/A6,3}$ and $SC_{Q/P,3}$ particle classes were further sorted via CIV focused 3D classification to find particles containing and missing CIV. R: Respirasome; $SC_{1,3}$: Supercomplex I+III_2; MA: membrane arm; PA: peripheral arm; $SC_{Q/P,3}$: Supercomplex with CI Q/P intermediate plus $CIII_2$, $SC_{Q/P/A6,3}$: Supercomplex with CI Q/P intermediate with NDUFA6, NDUFAB1-α and assembly factor NDUFAF2 plus $CIII_2$ (see Table S3).

bioRxiv preprint doi: https://doi.org/10.1101/2025.06.17.660237; this version posted June 18, 2025. The copyright holder for this preprint (which was not certified by peer review) is the author/funder, who has granted bioRxiv a license to display the preprint in perpetuity. It is made available under aCC-BY 4.0 International license.

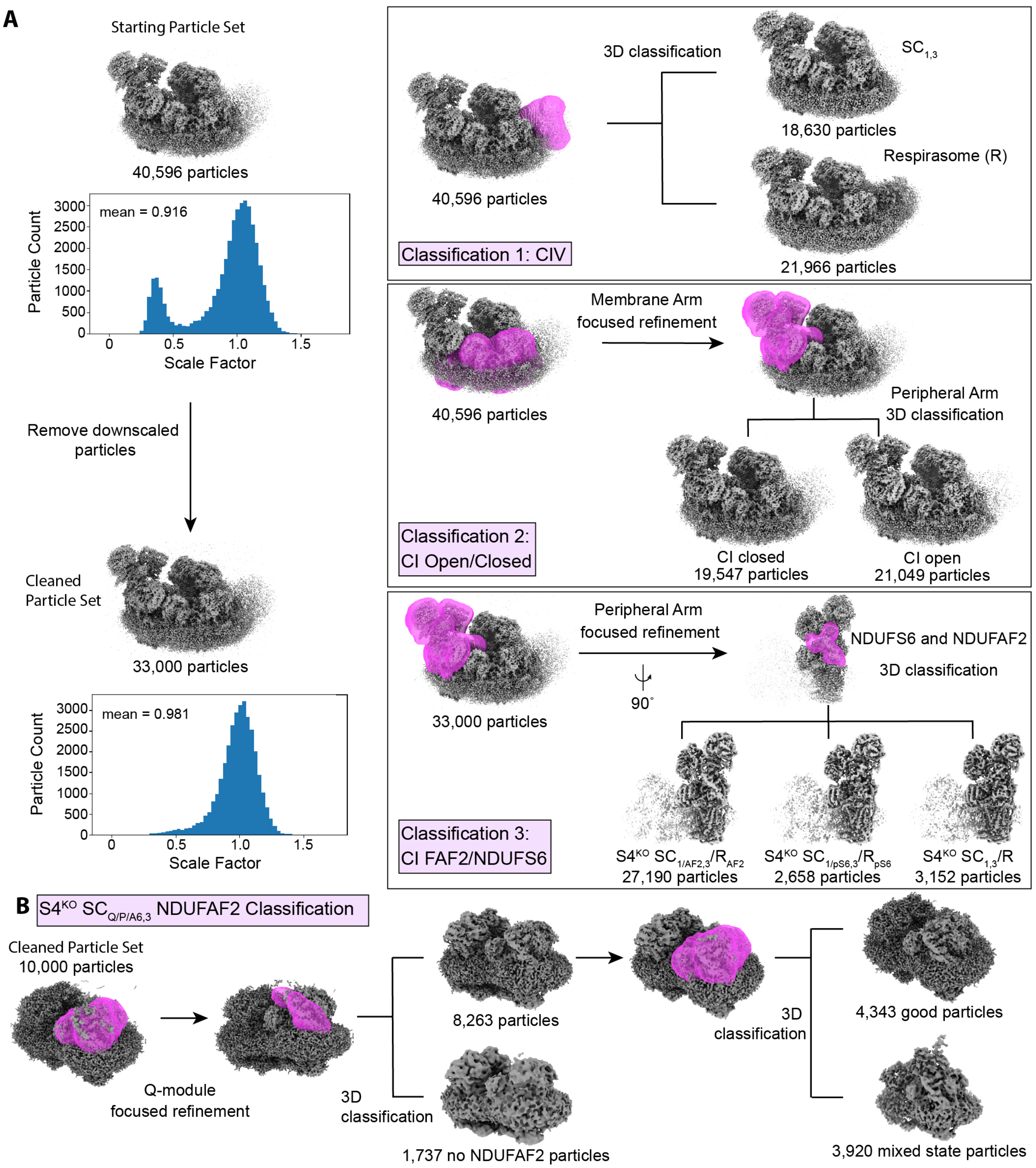

Figure S5. S4^{KO} additional classification strategy. (A) A S4^{KO} $SC_{1,3}$/R subset of 40,596 were sorted into $SC_{1,3}$ and R classes using a CIV focused 3D classification in cryoSPARC (classification 1). The 40,596 particles were also sorted into CI open and closed states by first doing a MA focused refinement followed by a PA 3D classification (classification 2). In parallel the 40,596 particles were cleaned by removing downscaled particles using a 3D Flex Data Prep job on cryoSPARC which yielded 33,000 good particles (left hand side). The clean set of 33,000

bioRxiv preprint doi: https://doi.org/10.1101/2025.06.17.660237; this version posted June 18, 2025. The copyright holder for this preprint (which was not certified by peer review) is the author/funder, who has granted bioRxiv a license to display the preprint in perpetuity. It is made available under aCC-BY 4.0 International license.

particles were used to sort particles into $SC_{1/AF2,3}$, $SC_{1/pS6,3}$ and $SC_{1,3}$ by first doing a PA focused refinement followed by a 3D classification using a NDUFS6 and NDUFAF2 mask (Classification 3). These three classifications defined sets of particles that were compared to generate the particle classes shown in Fig. S6. For example, the union between the $SC/R_{1/AF2,3}$, CI open and $SC_{1,3}$ classes defines the set of $SC_{1/AF2,3}$ open particles. **(B)** A clean set of 10,000 $SC_{Q/P/A6,3}$ particles aligned using a Q module mask followed by a NDUFAF2 focused 3D classification. This yielded 8,263 $SC_{Q/P/A6,3}$ particles and 1,737 $SC_{Q/P/A6,3\ noAF2}$ particles. An updated Q module mask was created and used for focused 3D classification which yielded the final $SC_{Q/P/A6,3}$ class of 4,343 good $SC_{Q/P/A6,3}$ particles and 3,920 junk particles. $SC_{1,3}$: Supercomplex I+III_2; R: Respirasome; CIV: Complex IV; CI: Complex I; MA: Membrane arm; PA: Peripheral arm; $SC_{1/AF2,3}$: Supercomplex I+III_2 with assembly factor NDUFAF2; $SC_{1/PS6,3}$: Supercomplex I+III_2 with partial NDUFS6 density; $SC_{Q/P,3}$: Supercomplex with CI Q/P intermediate plus $CIII_2$; $SC_{Q/P/A6,3}$: Supercomplex with CI Q/P intermediate with NDUFA6, NDUFAB1-α and plus $CIII_2$: $SC_{Q/P/A6,3\ noAF2}$ Supercomplex with CI Q/P intermediate with NDUFA6, NDUFAB1-α lacking NDUFAF2 and plus $CIII_2$ (see Table S3).

bioRxiv preprint doi: https://doi.org/10.1101/2025.06.17.660237; this version posted June 18, 2025. The copyright holder for this preprint (which was not certified by peer review) is the author/funder, who has granted bioRxiv a license to display the preprint in perpetuity. It is made available under aCC-BY 4.0 International license.

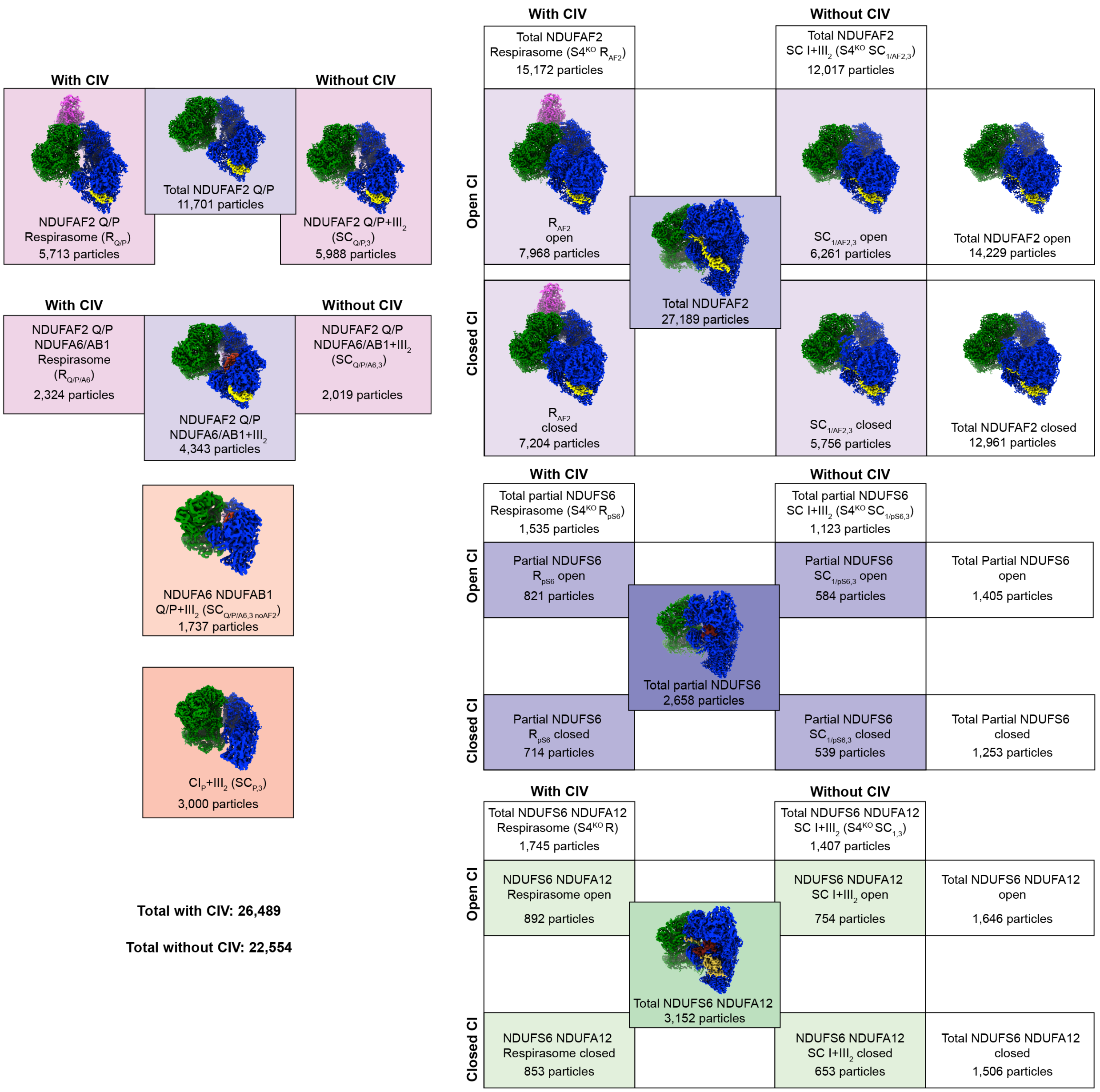

Figure S6. $S4^{KO}$ structural classes and particle distribution. Diagram outlining the different structures obtained from the $S4^{KO}$ sample through the classification strategy outlined in Fig. S5. Structures missing the N-module ($R_{Q/P}$, $SC_{Q/P,3}$, $SC_{Q/P/A6,3}$, $SC_{Q/P/A6,3\ noAF2}$, $SC_{P,3}$) are shown on the left. The $SC_{Q/P}$ and $SC_{Q/P/A6,3}$ classes are sorted into classes with and without CIV. Structures with the N-module (R_{AF2}, $SC_{1/AF2,3}$, R_{pS6}, $SC_{1/pS6,3}$, R and $SC_{1,3}$) are shown on the right. These particles were sorted in open and closed states and with and without complex IV by comparison of particle sets across the multiple classifications shown in Fig. S5A. The particle number is listed for each class. Reconstructions were obtained if shown in the box. We did not obtain reconstructions for classes with less than 1,000 particles. Complexes in reconstructions are colored with CI blue, $CIII_2$ green, CIV magenta, NDUFAF2 yellow, NDUFA6 red, NDUFS6 dark

bioRxiv preprint doi: https://doi.org/10.1101/2025.06.17.660237; this version posted June 18, 2025. The copyright holder for this preprint (which was not certified by peer review) is the author/funder, who has granted bioRxiv a license to display the preprint in perpetuity. It is made available under aCC-BY 4.0 International license.

red and NDUFA12 mustard. $SC_{1,3}$: Supercomplex I+III_2; R: Respirasome; CIV: Complex IV; CI: Complex I; MA: Membrane arm; PA: Peripheral arm; $SC_{1/AF2,3}$: Supercomplex I+III_2 with assembly factor NDUFAF2; $SC_{1/pS6,3}$: Supercomplex I+III_2 with partial NDUFS6 density; $SC_{Q/P,3}$: Supercomplex with CI Q/P intermediate plus $CIII_2$; $SC_{Q/P/A6,3}$: Supercomplex with CI Q/P intermediate with NDUFA6, NDUFAB1-α and plus $CIII_2$: $SC_{Q/P/A6,3\ noAF2}$ Supercomplex with CI Q/P intermediate with NDUFA6, NDUFAB1-α lacking NDUFAF2 and plus $CIII_2$ (see Table S3). $S4^{KO}$: NDUFS$4^{-/-}$ (see Table S3).

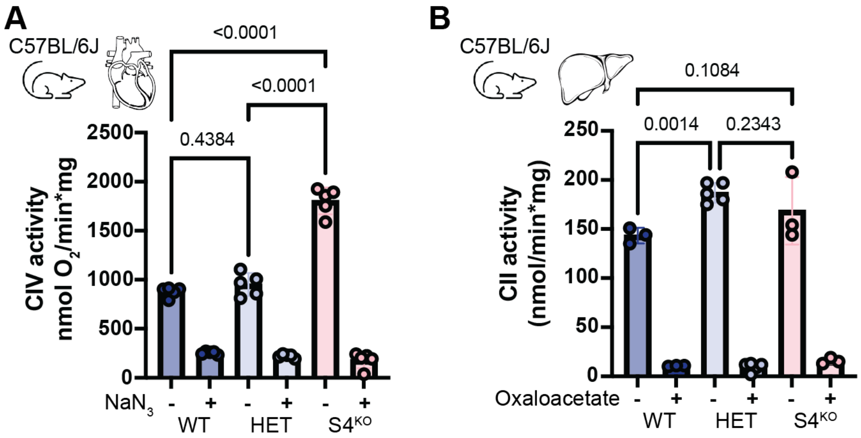

Figure S7 Heart CIV and liver CII functional data. (**A**) Murine heart maximal CIV oxygen consumption driven by excess ascorbate, TMPD and cyt *c*, n = 4-5, p-values from ordinary one-way ANOVA with multiple comparisons. (**B**) CII spectroscopic activity assay of murine liver mitochondrial membranes, n = 3-5, p-values from ordinary one-way ANOVA with multiple comparisons.

bioRxiv preprint doi: https://doi.org/10.1101/2025.06.17.660237; this version posted June 18, 2025. The copyright holder for this preprint (which was not certified by peer review) is the author/funder, who has granted bioRxiv a license to display the preprint in perpetuity. It is made available under aCC-BY 4.0 International license.

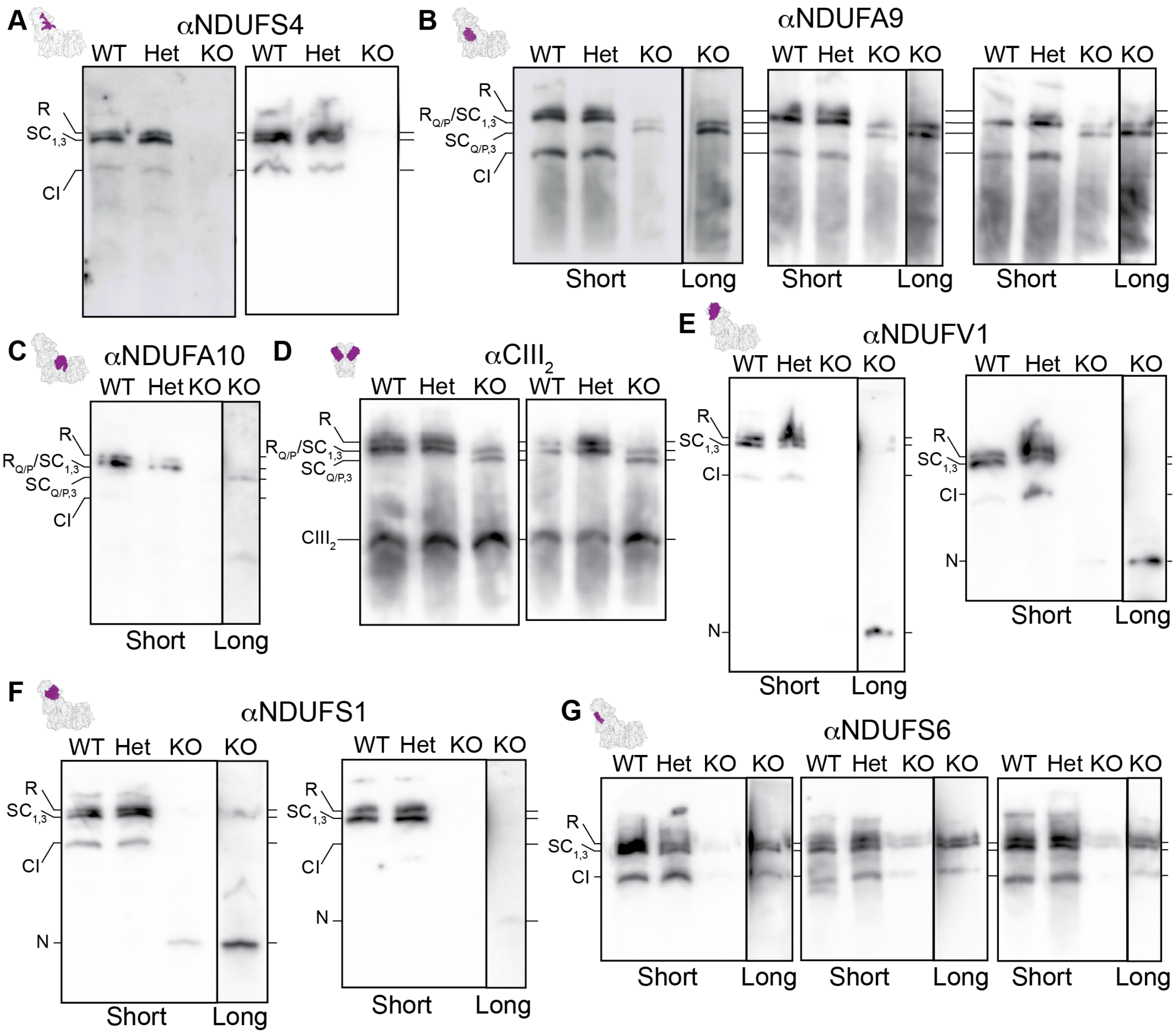

Figure S8. Additional Western Blots showing SCs containing CI assembly intermediates in the $S4^{KO}$ mouse liver mitochondria. Blue Native PAGE western blots of digitonin extracted mouse liver mitochondrial complexes using primary antibodies against (**A**) CI subunit NDUFS4, (**B**) CI subunit NDUFA9, (**C**) CI subunit NDUFA10, (**D**) CIII subunit UQCRC1, (**E**) CI subunit NDUFV1, (**F**) CI subunit NDUFS1 and (**G**) CI subunit NDUFS6. The location of each subunit is indicated in purple on the structure of the complex, top left of each panel. Each blot shown is an independent repeat. Short and long labels refer to the relative exposure times. Labels: WT: $NDUFS4^{+/+}$; Het: $NDUFS4^{+/-}$; $S4^{KO}$: $NDUFS4^{-/-}$; R: Respirasome; $SC_{1,3}$: Supercomplex I+III_2; CI: complex I; $R_{Q/P}$: Respirasome containing CI Q/P intermediate; $SC_{Q/P,3}$: Supercomplex Complex I Q/P intermediate with $CIII_2$. $CIII_2$: complex III dimer; N: N-module alone.

bioRxiv preprint doi: https://doi.org/10.1101/2025.06.17.660237; this version posted June 18, 2025. The copyright holder for this preprint (which was not certified by peer review) is the author/funder, who has granted bioRxiv a license to display the preprint in perpetuity. It is made available under aCC-BY 4.0 International license.

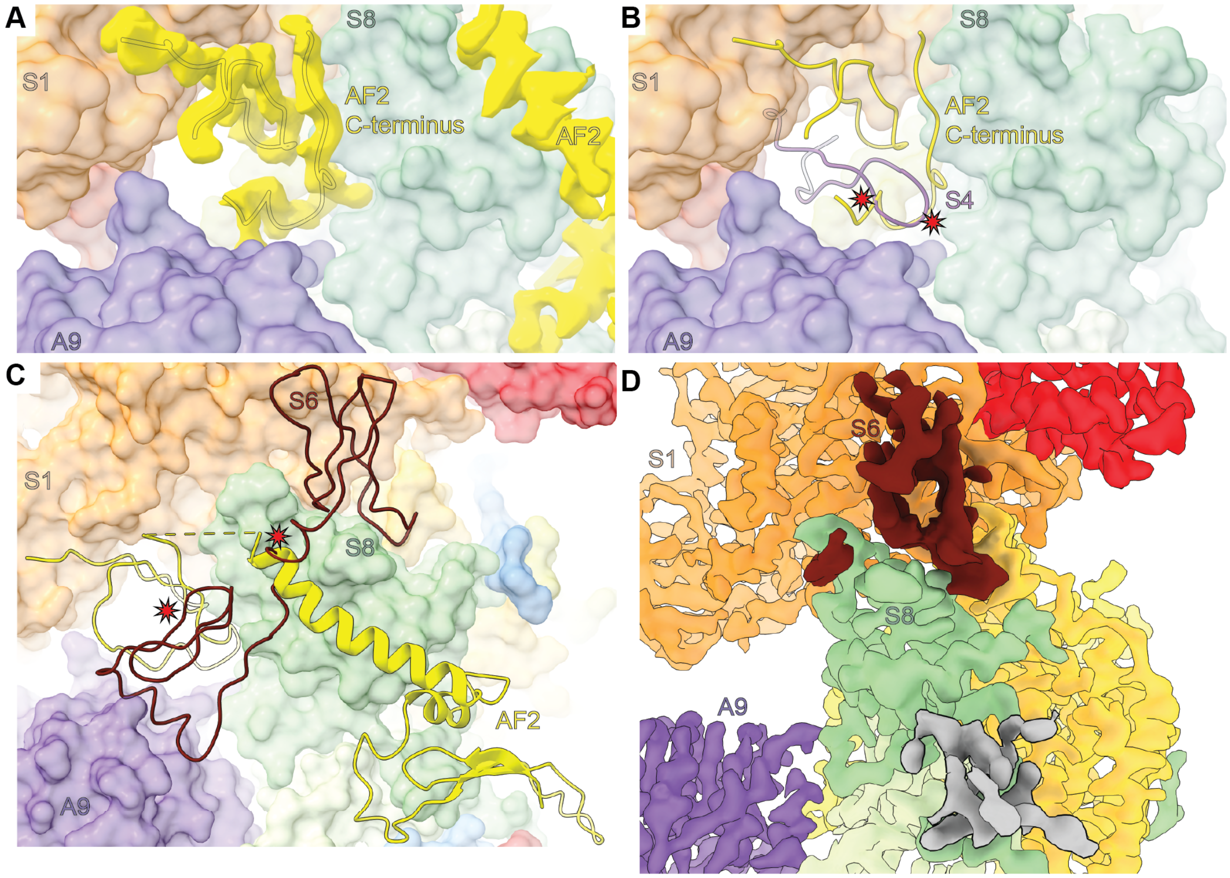

Figure S9. Comparison of NDUFAF2 binding to NDUFS4 and NDUFS6. (A) NDUFAF2 (AF2) density shown in yellow from $S4^{KO}$ closed $SC_{1/AF2,3}$. Density for AF2 allowed fitting of C-terminal residues $AF2^{P123-Y136}$ residues shown in cartoon in addition to the previously modeled residues, $AF2^{E141-E161}$ (*42*). Surfaces of NDUFS1 (S1), NDUFA9 (A9) and NDUFS8 (S8) are shown and labeled. **(B)** $S4^{KO}$ closed $SC_{1/AF2,3}$ structure shown with AF2 C-terminus in cartoon. NDUFS4 (S4) loop from WT closed $SC_{1,3}$ shown as purple cartoon. Red stars indicate where the AF2 C-terminus and S4 clash. S1, A9 and S8 are shown in surface and labeled. **(C)** $S4^{KO}$ closed $SC_{1/AF2,3}$ structure shown with AF2 as cartoons. NDUFS6 (S6) cartoon from WT closed $SC_{1,3}$ shown in dark red. Red stars indicate where AF2 and S6 clash. Surfaces of S1, A9 and S8 are shown and labeled. **(D)** $S4^{KO}$ $SC_{1/pS6,3}$ density shown colored by subunit. Partial S6 density is shown in dark red and extra density in the NDUFA12 region is shown in gray. This grey density was ambiguous and could not be modeled as either NDUFAF2 or NDUFA12 suggesting a disordered/mixed state. $SC_{1/AF2,3}$: Supercomplex I+III_2 with assembly factor NDUFAF2; $SC_{1,3}$: Supercomplex I+III_2; $SC_{1/PS6,3}$: Supercomplex I+III_2 with partial NDUFS6 density. WT: $NDUFS4^{+/+}$; $S4^{KO}$ $NDUFS4^{-/-}$ (see Table S3).

bioRxiv preprint doi: https://doi.org/10.1101/2025.06.17.660237; this version posted June 18, 2025. The copyright holder for this preprint (which was not certified by peer review) is the author/funder, who has granted bioRxiv a license to display the preprint in perpetuity. It is made available under aCC-BY 4.0 International license.

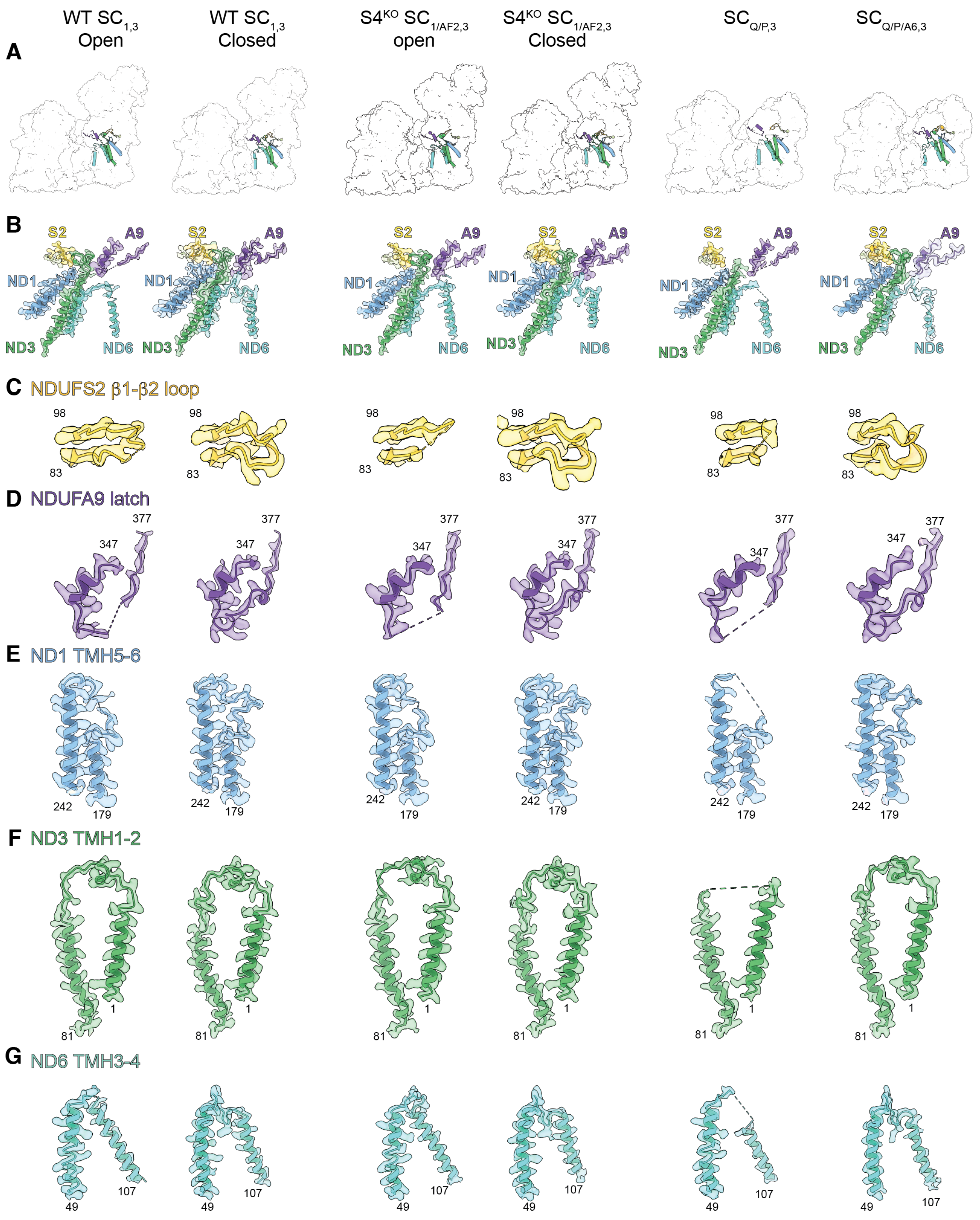

Figure S10. Structural features of active site loops across different states. (**A**) CI assembly for, from left to right, WT $SC_{1,3}$ Open, WT $SC_{1,3}$ Closed, $S4^{KO}$ $SC_{1/AF2,3}$ Open, $S4^{KO}$ $SC_{1/AF2,3}$ Closed, $SC_{Q/P,3}$ and $SC_{Q/P/A6,3}$ is shown as a transparent surface and the Q-site loops and the interface

bioRxiv preprint doi: https://doi.org/10.1101/2025.06.17.660237; this version posted June 18, 2025. The copyright holder for this preprint (which was not certified by peer review) is the author/funder, who has granted bioRxiv a license to display the preprint in perpetuity. It is made available under aCC-BY 4.0 International license.

forming loops are show as cartoons. (**B**) Zoomed-in view of the Q-site loops and the interface forming loops for, from left to right WT $SC_{1,3}$ Open, WT $SC_{1,3}$ Closed, $S4^{KO}$ $SC_{1/AF2,3}$ Open, $S4^{KO}$ $SC_{1/AF2,3}$ closed, $SC_{Q/P,3}$ and $SC_{Q/P/A6,3}$ show as cartoons embedded in the respective cryoEM density (**C**) CryoEM density map and model of NDUFS2 β1–β2 loop (aa 83 - 98) for, from left to right, WT $SC_{1,3}$ Open, WT $SC_{1,3}$ Closed, $S4^{KO}$ $SC_{1/AF2,3}$ Open, $S4^{KO}$ $SC_{1/AF2,3}$ Closed, $SC_{Q/P,3}$ and $SC_{Q/P/A6,3}$. (**D**) CryoEM density map and model of NDUFA9 latch (aa 347 - 377) for, from left to right, WT $SC_{1,3}$ Open, WT $SC_{1,3}$ Closed, $S4^{KO}$ $SC_{1/AF2,3}$ Open, $S4^{KO}$ $SC_{1/AF2,3}$ Closed, $SC_{Q/P,3}$ and $SC_{Q/P/A6,3}$. (**E**) CryoEM density map and model of ND1 TMH5-6 (aa 179 - 242) for, from left to right, WT $SC_{1,3}$ Open, WT $SC_{1,3}$ Closed, $S4^{KO}$ $SC_{1/AF2,3}$ Open, $S4^{KO}$ $SC_{1/AF2,3}$ closed, $SC_{Q/P,3}$ and $SC_{Q/P/A6,3}$. (**F**) CryoEM density map and model of ND3 TMH1-2 (aa 1- 81) for, from left to right, WT $SC_{1,3}$ Open, WT $SC_{1,3}$ Closed $SC_{1/AF2,3}$, $S4^{KO}$ $SC_{1/AF2,3}$ Open, $S4^{KO}$ $SC_{1/AF2,3}$ closed, $SC_{Q/P,3}$ and $SC_{Q/P/A6,3}$. (**G**) CryoEM density map and model of ND6 TMH3-4 (aa 49 -107) for, from left to right, WT $SC_{1,3}$ Open, WT $SC_{1,3}$ Closed, $S4^{KO}$ $SC_{1/AF2,3}$ Open, $S4^{KO}$ $SC_{1/AF2,3}$ closed, $SC_{Q/P,3}$ and $SC_{Q/P/A6,3}$G

bioRxiv preprint doi: https://doi.org/10.1101/2025.06.17.660237; this version posted June 18, 2025. The copyright holder for this preprint (which was not certified by peer review) is the author/funder, who has granted bioRxiv a license to display the preprint in perpetuity. It is made available under aCC-BY 4.0 International license.

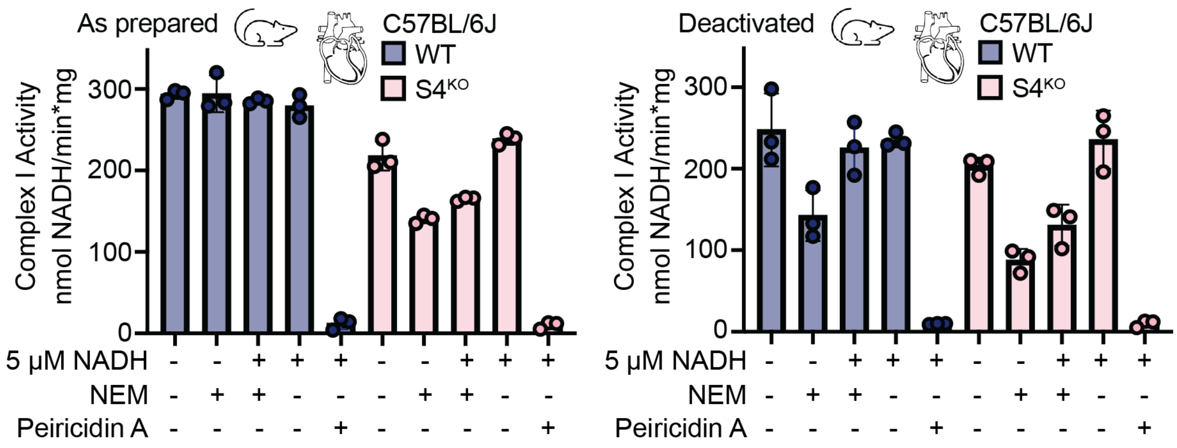

Figure S11. Functional characterization of the active and deactive states in the S4^{KO}.

Functional characterization of A-to-D transition from WT and S4^{KO} murine heart mitochondrial membranes as prepared (left) or deactivated (right) by measuring NADH oxidation at 340 nm. 2 mM N-ethylmaleimide (NEM), 5 µM NADH (pre activation) and 4 µM Piericidin A were used where indicated, n=3. WT: NDUFS4$^{+/+}$; S4^{KO}: NDUFS4$^{-/-}$.

bioRxiv preprint doi: https://doi.org/10.1101/2025.06.17.660237; this version posted June 18, 2025. The copyright holder for this preprint (which was not certified by peer review) is the author/funder, who has granted bioRxiv a license to display the preprint in perpetuity. It is made available under aCC-BY 4.0 International license.

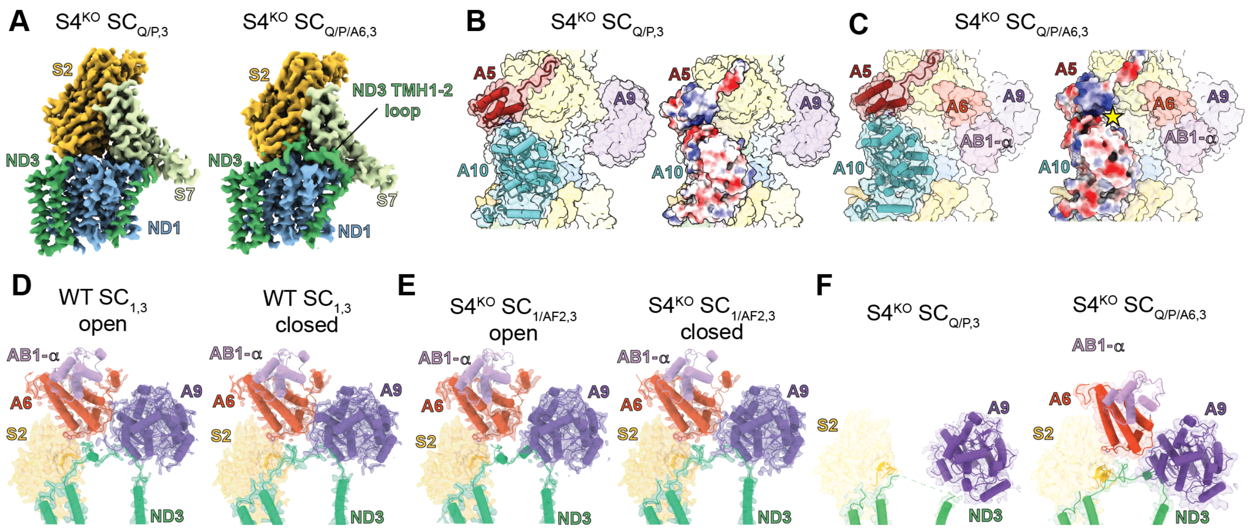

Figure S12. Comparison of $CI_{Q/P}$ and $CI_{Q/P/A6}$ structural features. (A) ND3 Density from $S4^{KO}$ $SC_{Q/P,3}$ (left) and $SC_{Q/P/A6,3}$ (right) colored by subunit, showing the ordering of the ND3 TMH1-2 loop after NDUFA6 binding. Other nearby subunits have been removed for clarity. **(B)** $S4^{KO}$ $SC_{Q/P,3}$ shown in transparent surface with NDUFA5 (A5) and NDUFA10 (A10) also shown in cartoon (left). Surface electrostatics for A5 and A10 shown (right). **(C)** $S4^{KO}$ $SC_{Q/P/A6,3}$ shown in transparent surface with A5 and A10 also shown in cartoon (left). Surface electrostatics for A5 and A10 shown (right). The star indicates the equivalent electropositive surface that would be impacted by the $NDUFA5^{R126Q}$ mutation in *C. elegans*. **(D)** Density from WT open $SC_{1,3}$ (left) and closed $SC_{1,3}$ (right) with cartoon overlay colored by subunit. **(E)** Density from $S4^{KO}$ open $SC_{1/AF2,3}$ (left) and $S4^{KO}$ closed $SC_{1/AF2,3}$ (right) with cartoon overlay colored by subunit. **(F)** Density from $S4^{KO}$ $SC_{Q/P,3}$ (left) and $SC_{Q/P/A6,3}$ (right) with cartoon overlay colored by subunit. $SC_{Q/P,3}$: Supercomplex CI Q/P intermediate plus $CIII_2$; $SC_{Q/P/A6,3}$: Supercomplex CI Q/P intermediate with NDUFA6, NDUFAB1-α plus $CIII_2$; $SC_{1,3}$: Supercomplex I+III_2; $SC_{1/AF2,3}$: Supercomplex I+III_2 with assembly factor NDUFAF2. NDUFS2: mustard; ND3: green; ND1: blue; NDUFS7: light green; NDUFA6: red-orange; NDUFA9: purple; NDUFAB1-α: lavender; NDUFA5: red; NDUFA:10 turquoise. WT: $NDUFS4^{+/+}$; $S4^{KO}$: $NDUFS4^{-/-}$.

bioRxiv preprint doi: https://doi.org/10.1101/2025.06.17.660237; this version posted June 18, 2025. The copyright holder for this preprint (which was not certified by peer review) is the author/funder, who has granted bioRxiv a license to display the preprint in perpetuity. It is made available under aCC-BY 4.0 International license.

Table S1 – Data collection and image processing

	Wild type (WT)	$S4^{KO}$
Microscope	Titan Krios (SLAC TEM-BETA)	
Voltage (kV)	300	
Camera	K3	
Data collection software	EPU	
Magnification	56,818	
Electron exposure (e/Å2)	50.5	49.78
Exposure time (s)	1.68	1.63
Frame number	40	40
Defocus range (µm)	1.0-1.8	1.0-1.8
Pixel size (Å)	0.86	0.86
Number of micrographs	10,570	958 16232
EMPIAR accession code	EMPIAR-12845	EMPIAR-12846

Table S2 – Model refinement statistics

	Wild type (WT)					$S4^{KO}$					
	$SC_{1,3}$ Closed	$SC_{1,3}$ Open	R Closed	R Open	$SC_{Q/P,3}$	$SC_{Q/P,3}$	$SC_{Q/P/A6,3}$	$SC_{1/AF2,3}$ Closed	$SC_{1/AF2,3}$ Open	$SC_{I/pS6,3}$	$SC_{1,3}$
Number of particles in final reconstruction	29,248	32,206	16,127	15,808	3,000	11,701	4,343	12,961	14,229	2,658	3,152
Resolution focused refinement (Å)	2.9 (2.8 - 3.0)	2.9 (2.8 - 3.1)	3.5 (3.0 - 3.5)	3.5 (3.0-3.5)	4.0 (3.8 -4.3)	3.5 (3.3 - 3.5)	3.9 (3.6 - 4.0)	3.2 (3.0 - 3.3)	3.3 (3.0-3.2)	3.9 (3.7 - 4.2)	3.7 (3.6 - 4.1)
EMDB accession code (EMD-)	71223 (71159, 71160, 71161, 71162, 71163)	71222 (71153, 71154, 71155, 71156, 71157)	71220 (71184, 71185, 71186, 71187, 71188)	71221 (71178, 71179, 71180, 71181, 71182)	71224 (71164, 71165, 71166, 71167, 71168)	71219 (71169, 71170, 71171, 71172)	71218 (71190, 71191, 71192, 71193)	71225 (71174, 71175, 71176, 71177, 71173)	71122 (71117, 71118, 71119, 71120, 71121)	71214 (71124, 71213, 71200, 71199)	71216 (71194, 71195, 71196, 71197)
**Model refinement**											
Model building software	Coot										
Model refinement software	Phenix										
Cross-correlation (mask)	0.85	0.86	0.84	0.85	0.78	0.87	0.86	0.86	0.88	0.87	0.88
Cross-correlation (volume)	0.82	0.84	0.81	0.82	0.77	0.85	0.84	0.83	0.85	0.85	0.87
**Model composition**											
Non-hydrogen atoms<br>Protein residues<br>Ligands	98210<br>11971<br>65	98106<br>11976<br>61	113160<br>13792<br>79	113252<br>13815<br>77	81815<br>9951<br>47	80598<br>9858<br>40	82118<br>10061<br>35	95769<br>11666<br>65	95719<br>11726<br>52	94823<br>11602<br>56	96618<br>11817<br>56
**Ramachandran**											
Favored (%)<br>Allowed (%)<br>Outlier (%)	95.70<br>4.27<br>0.03	95.53<br>4.39<br>0.08	94.76<br>5.13<br>0.10	94.13<br>5.78<br>0.10	95.69<br>4.19<br>0.11	94.38<br>5.52<br>0.10	94.16<br>5.63<br>0.21	96.06<br>3.91<br>0.03	95.23<br>4.66<br>0.11	95.91<br>3.96<br>0.13	95.63<br>4.25<br>0.12
Rotamer outliers	2.58	1.66	3.85	3.00	4.17	4.22	2.55	1.59	1.14	1.91	4.19
Clash score	2.78	2.86	3.83	3.64	7.96	3.87	11.29	2.85	4.38	8.25	6.85
**RMSD**											
Bond length (Å)	0.003	0.002	0.004	0.004	0.144	0.006	0.005	0.002	0.004	0.003	0.003
Bond angle (°)	0.534	0.555	0.645	0.676	3.007	0.714	0.861	0.468	0.707	0.839	0.685
**B factors (Å², min/max/mean)**											
Proteins	10.8/ 203.4/ 45.3	10.8/ 203.4/ 45.3	0.0/ 220.2/ 52.1	0.00/ 220.2/ 52.2	10.8/ 203.4/ 46.6	6.6/ 194.1/ 46.7	37.6/ 165.2/ 78.5	9.9/ 203.4/ 46.1	5.89/ 89.5/ 31.4	20.7/ 170.7/ 73.6	18.61/ 234.0/ 84
Ligands	11.9/ 192.35/ 44.06	11.9/ 192.35/ 45.96	11.9/ 192.3/ 46.4	11.9/ 206.9/ 51.0	11.9/ 192.3/ 48.7	11.9/ 192.3/ 53.4	50.9/ 112.3/ 78.9	11.9/ 192.4/ 43.8	11.02/ 73.76/ 34.88	34.3/ 121.9/ 70.6	27.20/ 169.28/ 91.54
MolProbity score	1.68	1.55	1.98	1.91	2.20	2.03	2.27	1.50	1.59	1.94	2.15
**Average atom inclusion**	0.8200	0.8690	0.8390	0.8030	0.6850	0.8010	0.7060	0.7350	0.8470	0.8180	0.7590
**Q-score**	0.5990	0.5820	0.57	0.5660	0.4470	0.5500	0.4930	0.5800	0.5740	0.5190	0.5270
PDB Accession code	9P30	9P2Z	9P2X	9P2Y	9P31	9P2W	9P2V	9P32	9PIL	9P2S	9P2T

bioRxiv preprint doi: https://doi.org/10.1101/2025.06.17.660237; this version posted June 18, 2025. The copyright holder for this preprint (which was not certified by peer review) is the author/funder, who has granted bioRxiv a license to display the preprint in perpetuity. It is made available under aCC-BY 4.0 International license.

Table S3 – Structural states for WT and S4^{KO} liver SCs

WT	CI state	Notes	CoQ binding site	Particle #	Shorthand
SC I+III$_2$	Open	Deactive CI	Disordered	32,206	SC$_{1,3}$
	Closed	Active CI	Ordered	29,248	
SC I+III$_2$+IV	Open	Respirasome, Deactive CI	Disordered	15,808	R
	Closed	Respirasome, Active CI	Ordered	16,127	
SC I$_{Q/P}$+III$_2$	Open	CI Q/P assembly intermediate missing the N-module (N-less), with assembly factor NDUFAF2 bound	Disordered	3,000	SC$_{Q/P,3}$

S4^{KO}	CI state	Notes	CoQ binding site	Particle #	Shorthand
SC I+III$_2$ NDUFS6 NDUFA12	Open	With NDUFS6 and NDUFA12, fully assembled	Too few particles	754	S4^{KO} SC$_{1,3}$
	Closed	With NDUFS6 and NDUFA12, fully assembled	Too few particles	653	
SC I+III$_2$+IV NDUFS6 NDUFA12	Open	Respirasome with NDUFS6 and NDUFA12, fully assembled	Too few particles	892	S4^{KO} R
	Closed	Respirasome with NDUFS6 and NDUFA12, fully assembled	Too few particles	853	
SC I+III$_2$ partial NDUFS6	Open	With partial NDUFS6 and only weak density in NDUFA12 site	Too few particles	584	S4^{KO} SC$_{1/pS6,3}$
	Closed	With partial NDUFS6 and only weak density in NDUFA12 site	Too few particles	539	
SC I+III$_2$+IV partial NDUFS6	Open	Respirasome with partial NDUFS6 and only weak density in NDUFA12 site	Too few particles	821	S4^{KO} R$_{pS6}$
	Closed	Respirasome with partial NDUFS6 and only weak density in NDUFA12 site	Too few particles	714	
SC I+III$_2$ NDUFAF2	Open	With assembly factor NDUFAF2	Disordered	6,261	S4^{KO} SC$_{1/AF2,3}$
	Closed	With assembly factor NDUFAF2,	Ordered	5,756	
SC I+III$_2$+IV NDUFAF2	Open	Respirasome with assembly factor NDUFAF2, Deactive CI	Disordered	7,968	S4^{KO} R$_{AF2}$
	Closed	Respirasome with assembly factor NDUFAF2, Active CI	Ordered	7,204	
SC I$_{Q/P}$+III$_2$	Open	CI Q/P assembly intermediate (N-less), with assembly factor NDUFAF2 bound	Disordered	5,988	SC$_{Q/P,3}$
SC I$_{Q/P}$+III$_2$+IV	Open	CI Q/P assembly intermediate (N-less), with assembly factor NDUFAF2 bound	Disordered	5,713	R$_{Q/P}$
SC I$_{Q/P+A6}$+III$_2$	Closed	CI Q/P assembly intermediate (N-less), with NDUFA6, NDUFAB1-a and assembly factor NDUFAF2 bound	Ordered	2,019	SC$_{Q/P/A6,3}$
SC I$_{Q/P+A6}$+III$_2$+IV	Closed	CI Q/P assembly intermediate (N-less), with NDUFA6, NDUFAB1-a and assembly factor NDUFAF2 bound	Ordered	2,324	R$_{Q/P/A6}$
SC I$_{Q/P+A6}$+III$_2$+IV no FAF2	Closed	CI Q/P (N-less), with NDUFA6, NDUFAB1-a and no NDUFAF2	Too few particles	1,737	R$_{Q/P/A6\ no\ AF2}$
SC I$_P$+III$_2$	N/A	CI proton-pumping P-module only	N/A	3,000	SC$_{P,3}$
